# Marginal Zinc Deficiency Aggravated Intestinal Barrier Dysfunction and Inflammation through ETEC Virulence Factors in a Mouse Model of Diarrhea

**DOI:** 10.3390/vetsci9090507

**Published:** 2022-09-16

**Authors:** Peng Wang, Qianqian Chen, Liping Gan, Xinyu Du, Qiyue Li, Hanzhen Qiao, Yinli Zhao, Jin Huang, Jinrong Wang

**Affiliations:** College of Biology Engineering, Henan University of Technology, Zhengzhou 450001, China

**Keywords:** marginal zinc deficiency, enterotoxigenic *Escherichia coli*, intestinal barrier, inflammation, virulence factors

## Abstract

**Simple Summary:**

Enterotoxigenic *Escherichia coli* (ETEC) is one of the most common bacterial causes of diarrhea in children and farm animals. Zinc has received widespread attention for its roles in the prevention and treatment of diarrhea. However, zinc is also essential for the pathogenesis of ETEC. This study aimed to explore the accurate effect and mechanisms of marginal zinc deficiency on ETEC k88 infection and host intestinal health. Using the newly developed marginal zinc deficiency and ETEC k88 infection mouse model, we found that marginal zinc deficiency aggravated growth impairment, diarrhea, intestinal morphology, intestinal permeability, and inflammation induced by ETEC k88 infection. Consistently, intestinal ETEC k88 shedding was also higher in mice with marginal zinc deficiency. However, marginal zinc deficiency failed to affect host zinc levels and correspondingly the zinc-receptor GPR39 expression in the jejunum. In addition, marginal zinc deficiency upregulated the relative expression of virulence genes involved in heat-labile and heat-stable enterotoxins, motility, cellular adhesion, and biofilm formation in the cecum content of mice with ETEC infection. These findings provide a new explanation for zinc treatment of ETEC infection.

**Abstract:**

Zinc is both essential and inhibitory for the pathogenesis of enterotoxigenic *Escherichia coli* (ETEC). However, the accurate effects and underlying mechanism of marginal zinc deficiency on ETEC infection are not fully understood. Here, a marginal zinc-deficient mouse model was established by feeding mice with a marginal zinc-deficient diet, and ETEC k88 was further administrated to mice after antibiotic disruption of the normal microbiota. Marginal zinc deficiency aggravated growth impairment, diarrhea, intestinal morphology, intestinal permeability, and inflammation induced by ETEC k88 infection. In line with the above observations, marginal zinc deficiency also increased the intestinal ETEC shedding, though the concentration of ETEC in the intestinal content was not different or even decreased in the stool. Moreover, marginal zinc deficiency failed to change the host’s zinc levels, as evidenced by the fact that the serum zinc levels and zinc-receptor *GPR39* expression in jejunum were not significantly different in mice with ETEC challenge. Finally, marginal zinc deficiency upregulated the relative expression of virulence genes involved in heat-labile and heat-stable enterotoxins, motility, cellular adhesion, and biofilm formation in the cecum content of mice with ETEC infection. These findings demonstrated that marginal zinc deficiency likely regulates ETEC infection through the virulence factors, whereas it is not correlated with host zinc levels.

## 1. Introduction

Diarrhea is one of the major health problems faced by humans and farm animals. More than 200 million children’s diarrhea cases have been reported globally [1,2], and more than 1,000,000 piglets have died from diarrhea in southern China [3]. Enterotoxigenic *Escherichia coli* (ETEC) is one of the most common bacterial causes of diarrhea in children and piglets [4,5]. ETEC infection-induced acute watery diarrhea caused rapid dehydration and was accompanied by gut barrier dysfunction and inflammation [6,7,8]. Two processes are required to initiate the ETEC infection. The first process is the colonization of ETEC on the intestinal epithelium mediated by colonization factors, mainly composed of fimbria and fimbria-related extracellular filamentous protein polymers [9]. Secondly, various kinds of enterotoxins, especially heat-labile (LT) and heat-stable enterotoxins (ST), are synthesized and released into intestinal epithelia, which further disrupt the intestinal electrolyte balance by stimulating water and electrolyte secretion into the intestinal lumen [10,11]. In addition, enterotoxins also elevate the relative expression of genes involved in inflammatory cytokines, such as *IL-1β*, in epithelial cells [12].

Zinc is a fundamental trace element and participates in various physiological processes, including catalyzing enzyme activity, cellular proliferation, and differentiation. Zinc deficiency is commonly associated with diarrhea. Zin deficiency has been estimated to account for 4% of diarrhea cases and mortality in children in developing countries [13]. Zinc is recommended by the World Health Organization (WHO) and The United Nations Children’s Fund for children below five years of age suffering with diarrhea [14]. These recommendations were based on the positive effect of zinc on intestinal barrier, immune response, and fluid transport [15,16,17]. Zinc can also improve growth performance and immune status and reduce diarrhea incidences in weaning piglets [18]. However, accumulating data have demonstrated that zinc is also essential for maintaining the pathogenic phenotype of ETEC. ETEC k88 needs to compete with the host for zinc under a restricted zinc environment, and inhibiting the zinc transporter results in ETEC growth perturbation [19]. In addition, marginal zinc was more common than severe zinc deficiency; many of the present studies regarding the relationship between ETEC and zinc deficiency have only used zinc depletion models [11], whereas data addressing the influence of marginal zinc deficiency on ETEC infection, host intestinal barrier, inflammation, and its mechanisms are lacking. In this study, we established a mouse diarrhea model by oral gavage ETEC k88 and explored the effect and mechanism of marginal zinc deficiency on ETEC k88 infection and host intestinal function.

## 2. Materials and Methods

### 2.1. Animals and Treatment

All studies on mice were approved by the Animal Care and Use Committee of the College of Biological Engineering, Henan University of Technology (Ethic Approval Code: Haut202110−5). All experiments were performed following the National Research Council’s Guide for the Care and Use of Laboratory Animals, Chinese Order No. 676 of the State Council, date 1 March 2017. A total of 36 female Institute for Cancer Research (ICR) mice (SPF grade) at 21 days old were purchased from Huafukang company (Beijing, China). All mice were individually raised in a temperature- and humidity-controlled room (temperature 21 ± 1 °C, humidity 50 ± 10%), and an artificial lighting schedule was provided from 08:00 to 20:00. Mice had free access to water and food.

After 7 days of acclimation, all mice were randomly assigned to a marginal zinc-deficient diet group (dZn), a marginal zinc-deficient diet and ETEC infection group (dZn + ETEC), a diet with normal levels of zinc group (Zn), or a diet with normal levels of zinc and ETEC infection group (Zn + ETEC) for 16 days, as a previous study showed serum and tissue zinc deficiency in mice fed the zinc-deficient diet for 14 days [20]. dZn and dZn + ETEC mice were both fed a marginal zinc-deficient diet, and Zn and Zn + ETEC mice were both fed a diet with normal zinc levels (marginal zinc-deficient diet plus 30 mg/kg zinc sulfate). The formulation and composition of the diets are shown in Table S1.

On day 12 of experimental diet feeding, mice were treated with streptomycin (0.5 g/L, Shanghai yuanye Co., Shanghai, China) together with fructose (6.7%, Solarbio Science & Technology Co., Beijing, China) in drinking water for 36 h to remove the gut commensal bacteria, according to previous studies [21,22]. Food was removed 12 h before inoculation. All the mice were administered intraperitoneally with Cimetidine (50 mg/kg, Sigma, St. Louis, MO, USA) 2–3 h prior to inoculation and then, were intragastrically gavaged with DMEM medium alone or 10^9^ colony-forming units (CFU) ETEC k88 resuspended in DMEM medium. The concentration of ETEC was determined by the relationship between OD value and CFU of ETEC. The serotype of ETEC was O149:K91:K88ac.

### 2.2. Samples

The criteria of diarrhea and diarrhea score were performed according to the previous study [22]. Briefly, the stool of mice was continuously monitored every 12 h, and mice stools with normal appearance, color change, wet tail or mucosa, and liquid stools were given scores of 0, 1, 2, and 3, respectively. The severity of diarrhea was determined by dividing the sum of all diarrhea scores by the number of mice. Mice were euthanized and sampled at 2 days post-infection after 8 h of fasting. The blood samples were collected and centrifuged at 3500× *g* for 15 min, and the serum samples were stored at −80 °C.

The jejunum was separated from the dZn, dZn + ETEC, dZn, and Zn + ETEC groups, and the middle part of the jejunum was carefully flushed with cold phosphate buffer and collected in the tube with 4% paraformaldehyde (Solarbio, Beijing, China). The tissue samples of jejunum and chyme samples of cecum were also collected and immediately preserved in liquid nitrogen for 2 h and then transferred and stored at −80 °C.

### 2.3. Intestinal Morphology

The fixed jejunum tissue was dehydrated with different concentrations of ethanol (100%, 95%, and 75%) and then embedded in paraffin wax. Transverse tissue samples of 5 μm were cut from the middle part of paraffin wax and were further stained with hematoxylin and eosin.

Villus height, crypt depth, and villus height/crypt depth were determined as previously described [23]. Briefly, a total of 10 crypt-villi units per transverse tissue samples were randomly measured; the villus height was calculated from the tip of the villus to the base, and the crypt depth was measured from the valley between villi to the basal membrane.

### 2.4. Inflammatory Cytokines

The serum inflammatory cytokines TNF-α, IL-6, and IL-1β were measured using the mouse-specific ELISA kit. The methods were performed according to the standard procedures of the protocol (Nanjing Jian Cheng Co., Ltd., Nanjing, China), with serum samples being diluted through saline if the measurements were out of the detection range. The thresholds of TNF-α, IL-6, and IL-1β were 1000 pg/mL, 600 pg/mL, and 200 pg/mL, respectively.

### 2.5. Intestinal Permeability

The permeability of the intestine was assessed by determining the serum levels of diamine oxidase (DAO), D-xylose, and endotoxin. The serum levels of DAO and D-xylose were measured using the mouse-specific kit (Nanjing Jian Cheng Bioengineering Co., Ltd., Nanjing, China) and performed according to the standard procedures of the protocol. The serum endotoxin level was determined by a limulus amebocyte lysate test (Xiamen Bioendo Technology Co., Ltd., Xiamen, China) and performed according to the standard procedures of the protocol. The serum levels of DAO, D-xylose, and endotoxin were calculated from the standard curve. Serum samples were be diluted through saline if the measurement was out of the detection range. The threshold of DAO, D-xylose, and endotoxin were 100 U/L, 60 nmol/mL, and 1.0 EU/mL, respectively.

### 2.6. Real-Time RT-PCR

All the primers used in this part were designed through primer BLAST from NCBI. The primer information is shown in Table S2. The total RNA of jejunum samples was extracted by using the RNAiso plus reagents, and the complementary DNA (cDNA) was further synthesized by using a reverse transcription kit (Vazyme, Nanjing, China). Real-time quantitative PCR (RT-qPCR) of cDNA was performed on a CFX96 Real-Time PCR Detection System (Analytik Jena, Jena, Germany) to quantify mRNA expression with a universal SYBR Green qPCR Master Mix (Vazyme, Nanjing, China). The RT-qPCR condition was shown as follows: 95 °C for 30 s, 40 cycles of 95 °C for 10 s, and 60 °C for 30 s, and a melt curve analysis with 95 °C for 15 s, 60 °C for 60 s, and 95 °C for 15 s. GAPDH was used as the internal control. The relative mRNA expression levels of jejunum samples of the dZn, dZn + ETEC, dZn, and Zn + ETEC groups were calculated by the 2^−ΔΔCT^ method, as previously described [24].

### 2.7. Stool ETEC Shedding and Tissue Burden

The ETEC in stool, cecum content, and jejunum was measured as previously described [25]. Briefly, the fresh stool, cecum content, and jejunum of mice at day 2 post-infection were collected and weighted in a sterile tube. All the samples were homogenized in saline and plated on MacConkey agar through gradient dilution. After 24 h incubation at 37 °C, colony-forming units (CFU) were counted. The concentration of *Escherichia coli* was expressed as the log CFU per gram of stool or tissue.

### 2.8. ETEC Growth Curve

The ETEC growth curve under different levels of zinc was determined as described [26]. Briefly, the equal OD_600_ value of ETEC resuspended in DMEM medium was added with different amounts of zinc sulfate to make the final concentration of zinc 0, 15, 30, and 60 mg/L. The bacteria were cultured at 37 °C and shaken at 180 r/min. The ETEC was continuously sampled at 0, 2, 4, 6, 8, 10, 12, 14, and 16 h and measured at OD_600_ by an ultraviolet spectrophotometer, and the timepoint of inoculation ETEC was regarded as 0 h. The ETEC growth curve was determined by the OD_600_ value.

### 2.9. Zinc Content

The serum zinc concentration was measured using a serum zinc measurement kit (Solarbio, Beijing, China), and the procedure was performed according to the kit’s instructions. The zinc contents in the diets were measured using flame atomic absorption spectrometry (ContrAA, Analytik Jena, Jena, Germany).

### 2.10. Virulence Factor Analysis

All primers used for virulence factor quantification are shown in Supplementary Table S3. The relative expression of the virulence factor in cecum content was determined the same as mRNA in the jejunum. Briefly, the total RNA of cecum content was obtained using the RNAiso plus reagents, and cDNA was further synthesized using a reverse transcription kit (Vazyme, Nanjing, China). qRT-PCR was performed to quantify mRNA expression with a universal SYBR Green qPCR Master Mix (Vazyme, Nanjing, China). The relative mRNA expression levels of the cecum content were calculated by the 2^−ΔΔCT^ method, and gapA was taken as the internal control.

### 2.11. Data Analysis

Data are expressed as means ± s.e.m. All data were analyzed by two-way ANOVA of GraphPad Prism (8.0.2 version, San Diego, CA, USA), and means were compared using the Student–Newman–Keuls test. *p* < 0.05 was considered statistically significant.

## 3. Results

### 3.1. Effect of Marginal Zinc Deficiency on Serum Zinc Concentration of Mice with ETEC Challenge

**Figure 1:**
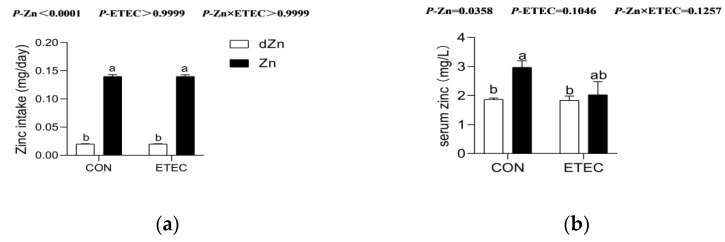
Effect of marginal zinc-deficiency on zinc intake and serum zinc levels of mice. (**a**) The zinc intake and (**b**) serum zinc levels in dZn, dZn + ETEC, Zn, and Zn + ETEC groups. *n* = 9/group. Data were expressed as means ± s.e.m; different superscript lowercase letters within each group indicate significantly different values (*p* < 0.05).

To explore the role of marginal zinc deficiency in mice with ETEC challenge, mice were fed a marginal zinc-deficient diet for 16 days and challenged with ETEC k88 at day 14 after antibiotics treatment [22]. Daily zinc intakes were higher (*p* < 0.05) in the Zn and Zn + ETEC groups compared with the dZn and dZn + ETEC groups (Figure 1a), though the body weight and average feed intake were not significantly different (Figure S1). In addition, the serum zinc levels were higher (*p* < 0.05) in the Zn group compared with the dZn group, which demonstrated that the marginal zinc deficiency mouse model was successfully established in the present study. It is noteworthy that marginal zinc deficiency failed to change the zinc levels in mice with ETEC challenge, as the serum zinc levels were not different (*p* > 0.05) between the dZn + ETEC and Zn + ETEC groups (Figure 1b).

### 3.2. Marginal Zinc-Deficient Mice Have Greater Diarrhea Scores and Body Weight Losses after ETEC Challenge

**Figure 2:**
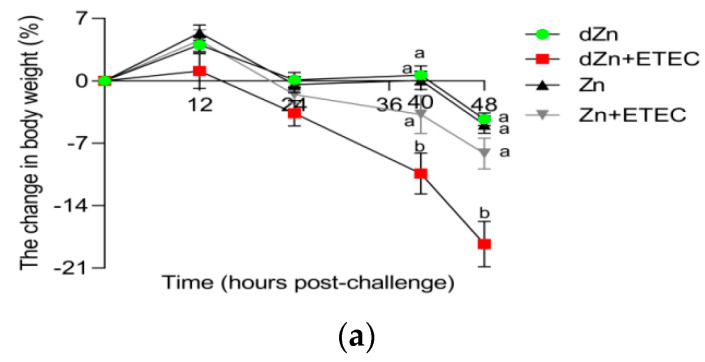

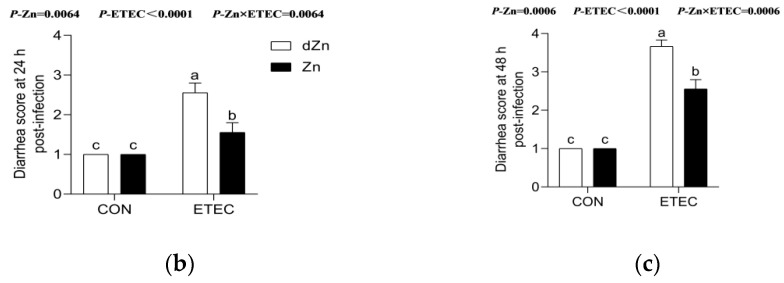
Effect of marginal zinc deficiency on the clinical symptoms induced by ETEC infection. (**a**) The effect of marginal zinc deficiency on the body weight change from 0 to 48 h after ETEC challenge. (**b**,**c**) The effect of marginal zinc deficiency on the diarrhea scores at 24 and 48 h after ETEC challenge. *n* = 9/group. Data were expressed as means ± s.e.m; different superscript lowercase letters within each group indicate significantly different values (*p* < 0.05).

Marginal zinc deficiency has no significant effect (*p* > 0.05) on body weight losses and diarrhea scores in mice without ETEC infection, whereas the body weight losses and diarrhea scores increased in mice with ETEC challenge. Compared with the Zn + ETEC group, dZn + ETEC mice showed higher (*p* < 0.05) body weight losses and diarrhea scores at 40 and 48 h post-ETEC infection, though these indicators were not significantly different (*p* > 0.05) between dZn and Zn groups (Figure 2a–c).

### 3.3. Intestinal Morphology Was Altered in Marginal Zinc-Deficient Mice with ETEC Challenge

**Figure 3:**
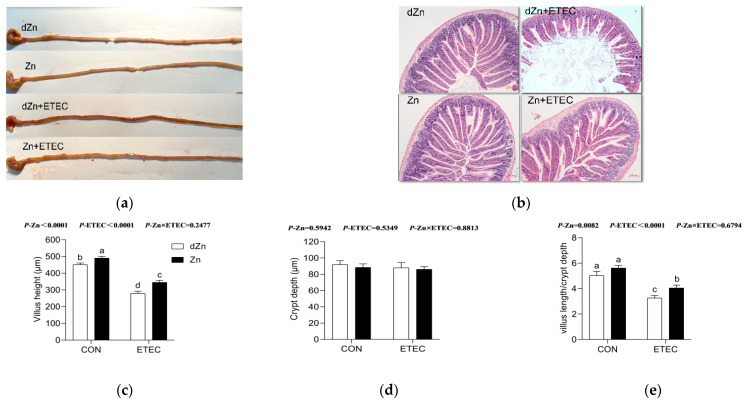
Effect of marginal zinc deficiency on the intestinal morphology of mice with ETEC infection. (**a**) The effect of marginal zinc deficiency on the intestinal morphology of mice after ETEC challenge. (**b**) The effect of marginal zinc deficiency on the jejunum with hematoxylineosin staining with the magnification factor of 100×. (**c**–**e**) The effect of marginal zinc deficiency on the villus height, crypt depth, and villus height/crypt depth. *n* = 9/treatment. Data were expressed as means ± s.e.m; different superscript lowercase letters within each group indicate significantly different values (*p* < 0.05).

Intestinal morphology was analyzed by the villus morphology and histopathology in the jejunum, the main tissue responsible for nutrients absorption. Compared with the Zn group, the dZn group showed slight changes in the jejunum morphology, except for a lower villus height (Figure 3). Conversely, marginal zinc deficiency aggravated the intestinal morphology damage caused by ETEC infection. Compared with the Zn + ETEC group, dZn + ETEC mice showed obvious bleeding points, impaired jejunum villus, and lower villus height and villus height/crypt depth of the jejunum (Figure 3a–e).

### 3.4. Marginal Zinc Deficiency Decreased Host Defense against ETEC Infection Induced Disruption of Intestinal Barrier and Permeability

**Figure 4:**
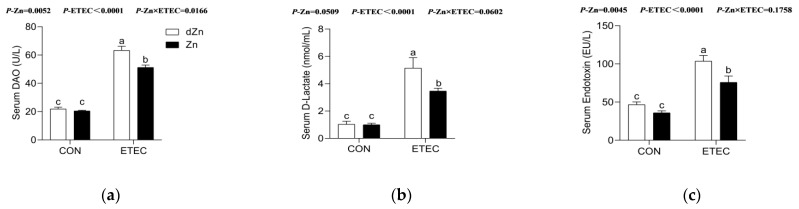
Effect of marginal zinc deficiency on the intestinal permeability of mice with ETEC infection. The effect of marginal zinc deficiency on serum levels of (**a**) DAO, (**b**) D-Lactate, and (**c**) endotoxin in serum of mice with ETEC challenge. *n* = 9/treatment. Data were expressed as means ± s.e.m; different superscript lowercase letters within each group indicate significantly different values (*p* < 0.05).

The intestinal barrier and its permeability are vital for the host to fight against endogenous and exogenous hazardous substances. Serum diamine oxidase (DAO) and D-lactate are often regarded as essential indicators of intestinal barrier function. Marginal zinc deficiency has no significant effect (*p* > 0.05) on serum levels of DAO and D-lactate in mice without ETEC challenge, whereas it increased (*p* < 0.05) the serum levels of DAO and D-lactate in mice with ETEC challenge (Figure 4a,b). Endotoxin was mainly synthesized by ETEC and released into a host intestinal epithelium [10]. The decreased serum endotoxin in dZn + ETEC compared with Zn + ETEC (Figure 4c) also verified the disruption of intestinal barrier function and permeability.

**Figure 5:**
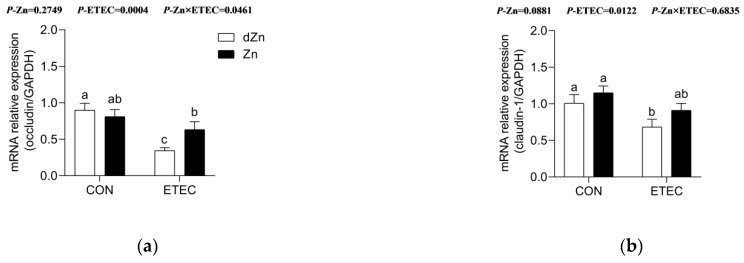

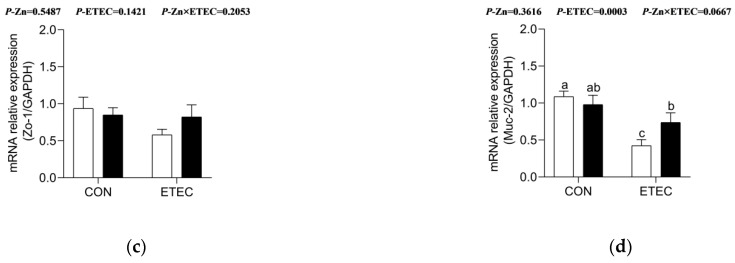
Effect of marginal zinc deficiency on the relative expression of mRNAs involved in intestinal barrier function of mice with ETEC infection. The effect of marginal zinc deficiency on the relative expression of (**a**) occludin, (**b**) claudin-1, (**c**) Zo-1, and (**d**) Muc-2 in the jejunum of mice with ETEC challenge. *n* = 9/treatment. Data were expressed as means ± s.e.m; different superscript lowercase letters within each group indicate significantly different values (*p* < 0.05).

To further explore the role of marginal zinc deficiency on the intestinal function of ETEC-challenged mice, the relative expression of mRNAs responsible for intestinal barrier was analyzed. Compared with the Zn group, dZn mice showed no significant effect (*p* > 0.05) on the relative expression of occludin, claudin-1, Zo-1, and Muc-2. However, marginal zinc deficiency aggravated the intestinal barrier function injury caused by ETEC infection, as dZn + ETEC group mice exhibited lower (*p* < 0.05) levels of occludin, claudin-1, Zo-1, and Muc-2 compared with the Zn + ETEC group (Figure 5a–d).

### 3.5. Marginal Zinc Deficiency Aggravated Intestinal Inflammation in Mice with ETEC Challenge

**Figure 6:**
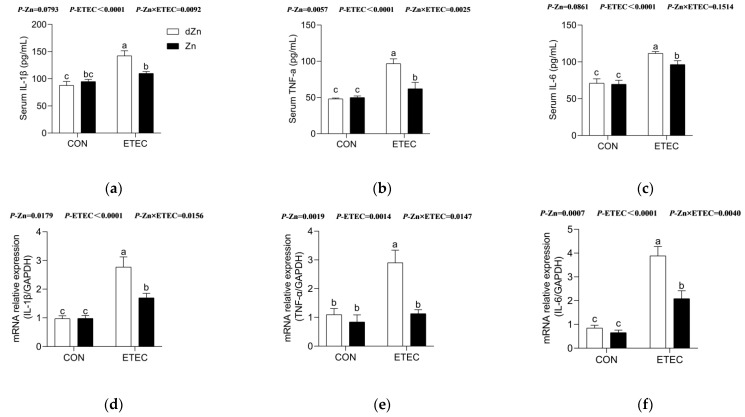
Effect of marginal zinc deficiency on the intestinal inflammation of mice with ETEC infection. The effect of marginal zinc deficiency on serum levels of (**a**) IL-1β, (**b**)TNF-α, and (**c**) IL-6 in serum of mice with ETEC challenge. The effect of marginal zinc deficiency on the relative expression of (**d**) *IL-1β*, (**e**)*TNF-α*, and (**f**) *IL-6* in jejunum of mice with ETEC challenge. *n* = 8/treatment. Data were expressed as means ± s.e.m; different superscript lowercase letters within each group indicate significantly different values (*p* < 0.05).

In addition to the intestinal barrier and its permeability, marginal zinc deficiency also induced a more powerful intestinal inflammatory reaction in mice with ETEC challenge. Marginal zinc deficiency caused higher (*p* < 0.05) serum levels of inflammatory cytokines (IL-1β, TNF-α, and IL-6) and intestinal mRNAs expression of inflammatory cytokines (*IL-1β*, *TNF-α,* and *IL-6*) in the dZn + ETEC group compared with the Zn + ETEC group, though no significant differences (*p* > 0.05) were found between the dZn and Zn groups (Figure 6a–f).

### 3.6. Marginal Zinc Deficiency Aggravated Intestinal Injury Was Associated with NF-κB

**Figure 7:**
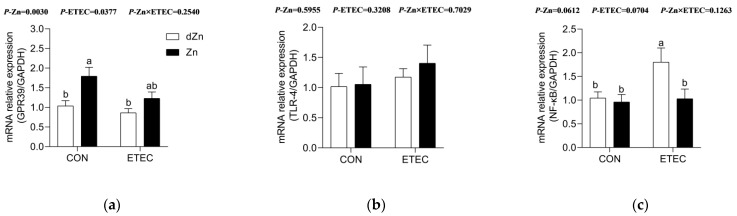
Effect of marginal zinc deficiency on the relative expression of *GPR39*, *TLR4,* and *NF-κB* in the jejunum. Marginal zinc deficiency altered the relative expression of mRNAs of (**a**) *GPR39*, (**b**) *TLR4*, and (**c**) *NF-κB* in the jejunum of mice with ETEC challenge. *n* = 9/treatment. Data were expressed as means ± s.e.m; different superscript lowercase letters within each group indicate significantly different values (*p* < 0.05).

The G protein-coupled receptor 39 (GPR39) has been recognized as a critical zinc-sensing receptor, which plays an essential role in intestinal barrier function [18]. Marginal zinc deficiency downregulated (*p* < 0.05) the relative expression of *GPR39* in the jejunum of mice without ETEC challenge, whereas it had no significant effect in the jejunum of mice with ETEC challenge (Figure 7a). Nuclear factor kappa-B (NF-κB), the key transcription factor of pro-inflammatory genes, promotes immunity by controlling the expression of genes involved in inflammation [27]. In this study, marginal zinc deficiency increased (*p* < 0.05) the relative expression of *NF-κB*, though *TLR-4* was not significantly different in mice with ETEC infection (Figure 7b,c). Marginal zinc deficiency aggravating the intestinal injury in mice was likely to be associated with NF-κB, but it did not correlate with the zinc-receptor GPR39.

### 3.7. Marginal Zinc Deficiency Altered Anion Transporters in Mice with ETEC Challenge

**Figure 8:**
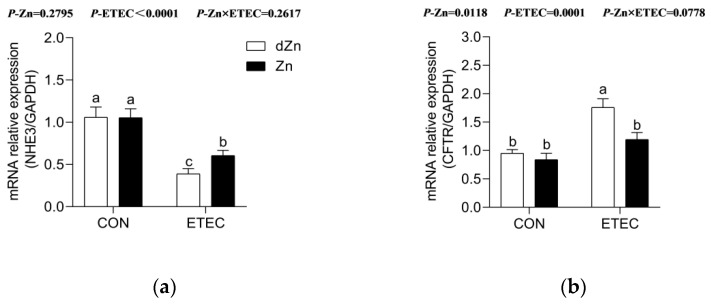
Effect of marginal zinc deficiency on the relative expression of mRNAs involved in ion absorption and secretion after ETEC infection. Marginal zinc deficiency altered the relative expression of mRNAs of (**a**) *NHE3* and (**b**) *CFTR* in the jejunum of mice with ETEC challenge. *n* = 9/treatment. Data were expressed as means ± s.e.m; different superscript lowercase letters within each group indicate significantly different values (*p* < 0.05).

Na^+^/H^+^ exchange protein 3 (NHE3) and cystic fibrosis transmembrane conductance regulator (CFTR) mediate the Na^+^ absorption and anion secretion into the intestine, respectively [28]. Marginal zinc deficiency alone has no significant effect on the relative expression of *NHE3* and *CFTR* in jejunum (Figure 8a,b). Compared with the Zn + ETEC group, the dZn+ETEC group showed lower relative expression of *NHE3*, but higher (*p* < 0.05) *CFTR* in the jejunum, implying that marginal zinc deficiency aggravated intestinal anion secretion and inhibited Na^+^ absorption in mice with ETEC challenge.

### 3.8. Marginal Zinc Deficiency Increased the ETEC Shedding in the Jejunum of Mice with ETEC Challenge

**Figure 9:**
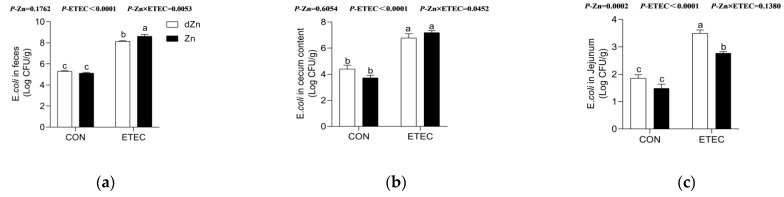
Effect of marginal zinc deficiency on the ETEC shedding in the intestine. Marginal zinc deficiency altered the ETEC content in (**a**) feces, (**b**) cecum content, and (**c**) jejunum in mice with ETEC challenge. *n* = 9/treatment. Data were expressed as means ± s.e.m; different superscript lowercase letters within each group indicate significantly different values (*p* < 0.05).

The enterotoxins LT and ST have been demonstrated to be the leading causes of impaired intestinal barriers and anion transporters in response to ETEC infection [10]. ETEC in stool, cecum content, and jejunum were measured to reveal the role of marginal zinc deficiency on ETEC shedding. Zinc deficiency has no significant effect (*p* > 0.05) on the concentration of ETEC in stool, cecum content, and jejunum of mice without ETEC challenge (Figure 9a–c). Consistently, marginal zinc deficiency also showed no significant effect (*p* > 0.05) on the concentration of ETEC in the cecum content of mice with ETEC infection, though the ETEC in stool was lower (*p* < 0.05) in the dZn + ETEC group compared with the Zn + ETEC group. Surprisingly, marginal zinc deficiency significantly increased (*p* < 0.05) the concentration of ETEC in the jejunum in mice with ETEC challenge (Figure 8c); these results implied that the higher jejunum ETEC shedding in marginal zinc-deficient mice is not likely to be associated with ETEC in intestinal content, though the ETEC growth was inhibited in vitro by the same zinc levels as in vivo (Supplemental Figure S2).

### 3.9. Marginal Zinc Deficiency Enhanced Virulence Factors in Mice with ETEC Infection

**Figure 10:**
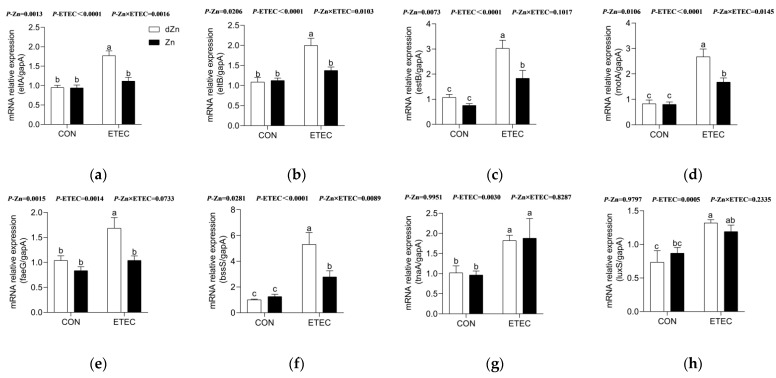
Effect of marginal zinc deficiency on the ETEC virulence in mice. Marginal zinc deficiency altered the relative repression levels of virulence genes involved in (**a**,**b**) heat-labile toxins (eltA and eltB), (**c**) heat-stable toxin (estB), (**d**) motility (motA), (**e**) cellular adhesion (faeG), (**f**,**g**) biofilm formation (bssS and tnaA), and (**h**) quorum sensing (luxS). *n* = 9/treatment. Data were expressed as means ± s.e.m; different superscript lowercase letters within each group indicate significantly different values (*p* < 0.05).

To further explore the mechanism accounting for intestinal injury and ETEC shedding in marginal zinc-deficient mice, mRNAs involved in LT, ST, motility, cellular adhesion, biofilm formation, and quorum sensing were analyzed. Marginal zinc deficiency has no significant effect (*p* > 0.05) on the relative expression of virulence genes in control mice. However, marginal zinc deficiency significantly increased (*p* < 0.05) the relative expression of genes involved in LT (*eltA* and *eltB*), ST (*estB*), motility (*motA*), cellular adhesion (*faeG*), and biofilm formation (*bssS*) in mice with ETEC infection, though no significant differences (*p* > 0.05) were found in the relative expression of genes involved in quorum sensing (*luxS*) (Figure 10a–h).

## 4. Discussion

Zinc has received widespread attention for its role in the prevention, control, and treatment of diarrhea in both children and farm animals. However, limited information about the effect of zinc deficiency, especially marginal zinc deficiency, on ETEC k88 infection bas been acquired. Here, a mouse diarrhea model was established by oral gavage ETEC k88 after the gut commensal microbiota were disrupted by antibiotics, and a marginal zinc-deficient mouse model was also established by feeding mice a marginal zinc-deficient diet. Consistent with the symptoms of piglet diarrhea models [29,30], ETEC k88-infected mice also exhibited more significant body weight losses, diarrhea scores, and intestinal barrier damage and inflammatory reactions. In addition, marginal zinc-deficient mice exhibited severe symptoms of serum zinc deficiency, which is in agreement with previous studies on mice [31].

The intestinal epithelium functions as a barrier between the external environment and the closely regulated internal milieu, and the increased intestinal permeability was commonly associated with gut injury [32]. Zinc deficiency has been demonstrated to decrease the ability of zinc absorbed into the host [6] and further induces intestinal tight junction injury and inflammatory reaction [18,33]. In this study, marginal zinc deficiency aggravated the intestinal injury, including decreased villus height, villus height/crypt depth, and relative expression of genes responsible for tight junction and mucin protein, whereas it increased intestinal permeability and pro-inflammatory cytokines. GPR39 is a zinc-receptor, which is expressed ubiquitously throughout the gastrointestinal tract, especially in intestinal epithelial cells. GPR39 has a dual role in promoting the proliferation of intestinal epithelial cells and the expression of tight junctional proteins [34]. In accordance with decreased zinc intake, marginal zinc deficiency also reduced serum zinc levels and the relative expression of *GPR39* in ETEC uninfected mice. Correspondingly, the relative expression of *GPR39* was not different between the dZn + ETEC and Zn + ETEC groups, as serum zinc levels were not changed between these two groups. These observations suggested marginal zinc aggravated intestinal barrier dysfunction was not likely associated with the host’s zinc levels.

Toll-like receptor 4 (TLR4) is the best-characterized pattern recognition transmembrane receptor, which can recognize many exogenous substances, such as endotoxin or lipopolysaccharide (LPS) from Gram-negative bacteria, and then initiate inflammatory response through the TLR4/NF-κB signaling pathway [35]. In this study, ETEC infection induced higher serum levels of endotoxin, correspondingly higher levels of serum pro-inflammatory cytokines, and higher relative expressions of mRNAs involved in pro-inflammatory cytokines in the jejunum, though the relative expression of *TLR4* was not different. Consistently, marginal zinc deficiency induced higher serum levels of endotoxin, and thus higher serum pro-inflammatory cytokines, and the relative expression of intestinal mRNAs accounted for pro-inflammatory cytokines.

The colonization of ETEC on the intestinal epithelium and subsequent enterotoxin release are two vital processes in the pathogenesis of ETEC infection [10]. Consistent with common pathogens of enteroaggregative *Escherichia coli* [26], ETEC k88 cultured in the zinc-deficient medium exhibited higher growth speed and biomass in vitro, compared with the zinc-supplemented group. Moreover, marginal zinc deficiency aggravated the colonization of ETEC k88 on the intestinal epithelium, as evidenced by the result that ETEC k88 shedding in the intestine was higher in the dZn + ETEC group compared with the Zn + ETEC group. However, marginal zinc deficiency failed to affect the concentration of ETEC in the cecum content and even decreased the concentration of ETEC k88 in the stool when ETEC co-treated with zinc. The growth-promoting effect of zinc on ETEC k88 was likely associated with its essential role in ETEC k88 growth. ETEC k88 commonly competes with a host for zinc under a restricted zinc environment, and inhibiting the zinc transporter results in ETEC k88 growth perturbation [19]. Consistently, zinc supplementation increased the concentration of ETEC H10407 in stool under a zinc-restricted environment [6]. FaeG, the major component of k88(+) fimbriae, contributes to the pathogen colonization of the intestinal epithelium [36,37]. Our results showed that marginal zinc deficiency upregulated the relative expression of *FaeG* in intestinal content, along with genes involved in cellular motility and biofilm formation, which partly explains the increased intestinal ETEC colonization in ETEC-infected mice. Enterotoxins, especially LT and ST, stimulated water and electrolyte secretion in the intestinal lumen through Na^+^/H^+^ exchange protein 3 (NHE3) and the cystic fibrosis transmembrane conductance regulator (CFTR), thus leading to diarrhea [11,38]. Our results also showed that marginal zinc deficiency upregulated the relative expression of *eltA* and *eltB*, responsible for LT, and *estB*, responsible for ST, in ETEC-infected mice. In line with the elevated ST and LT, marginal zinc deficiency downregulated the relative expression of intestinal *NHE3,* whereas it upregulated *CTFR* in ETEC-infected mice. These findings might provide a new regulation mechanism of marginal zinc deficiency in ETEC infection.

## 5. Conclusions

Taken together, the findings of this study demonstrated that marginal zinc deficiency aggravated the growth impairment, intestinal morphology, barrier function, and inflammation induced by ETEC infection. The regulatory role of marginal zinc deficiency was highly correlated with virulence factors of ETEC in vivo, but not with host zinc levels. Further study is warranted to explore the potential mechanisms of marginal zinc deficiency in virulence factors of ETEC k88 in farm animals.

## Data Availability

Not applicable.

## Supplementary Materials

The following supporting information can be downloaded at: https://www.mdpi.com/article/10.3390/vetsci9090507/s1, Figure S1: Zinc deficiency altered the body weight, feed intake, and zinc intake of mice; Figure S2: Effect of zinc concentration on the growth curve of ETEC k88; Table S1: Ingredients and composition of marginal zinc-deficient diet and normal levels of zinc diet; Table S2: Primer set for real-time RT-PCR analysis; Table S3: Primer set for virulence factors analysis.
