# Supplementary material for: Marginal Zinc Deficiency Aggravated Intestinal Barrier Dysfunction and Inflammation through ETEC Virulence Factors in a Mouse Model of Diarrhea
Source: Vet Sci. 2022 Sep 16;9(9):507. doi: 10.3390/vetsci9090507 (PMC9503546; doi:10.3390/vetsci9090507)
Supplement: Supplementary file 1 [file vetsci-09-00507-s001.zip › vetsci-1862943-supplementary.pdf]

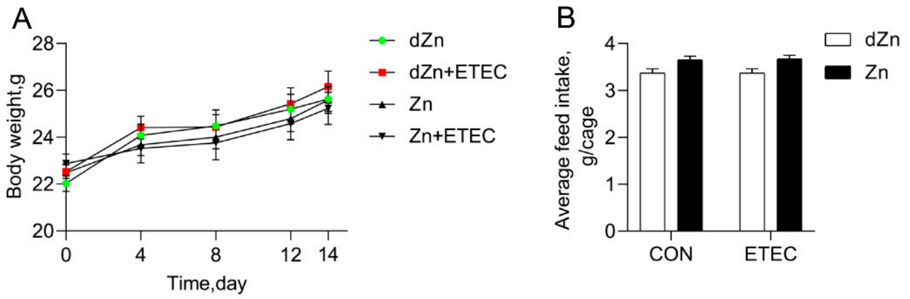

Supplementary Figure S1. Marginal zinc deficiency altered the body weight, and average feed intake of mice. Effect of marginal zinc deficiency on body weight (A) and average feed intake (B) of mice.

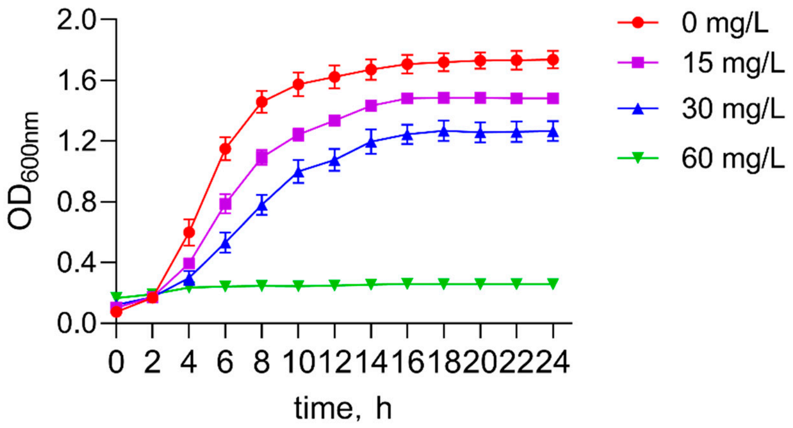

Supplementary Figure S2. Effect of zinc concentration on the growth curve of ETEC k88.

Supplementary Table S1. Ingredients and composition of marginal zinc deficient diet and normal levels of zinc diet

Items	Marginal zinc deficient diet	Normal levels of zinc diet
Ingredients, %		
Egg White, Spray Dried	20.0	20.0
Corn starch	15.0	14.9865
Sucrose	50.26	50.26
Cellulose	5.00	5.00
Corn oil	5.00	5.00
Biotin	0.04	0.04
Vitamin premix*	1.00	1.00
Minerals premix#	3.50	3.50
Choline bitartrate	0.20	0.20
Zinc sulfate	0.00	0.0135
Total	100	100
Nutrients content, %		
Carbohydrate	66.0	66.0
Fat	5.00	5.00
Protein	20.0	20.0
Zinc levels, mg/kg$		
Marginal zinc deficient diet	7.00	39

*Diet per kilograms: 4,000 IU VA, 1,000 IU VD_3, 750 IU VE, 0.75 mg VK, 6 mg VB_1, 6 mg VB_2, 7 mg VB_6, 0.025 mg VB_{12}, 30 mg nicotinic acid, 16 mg pantothenic acid, 2 mg folic acid and 0.2 mg biotin.

Diet per kilograms: 5 g Ca,1.56 g P, 3.6 g K, 0.3 g S, 0.5 g Mg, 6 mg Cu, 37 mg Fe, 10.5 mg Mn, 0.2 mg Se, 0.2 mg I.

$ The zinc levels in diet measured by flame atomic absorption spectrometry.

Supplementary Table S2. Primer set for real-time RT-PCR analysis

Gene	5'–3' Primer sequence
Gapdh	F:TTGAGGTCAATGAAGGGGTC
	R:TCGTCCCGTAGACAAAATGG
Occludin	F:TGGCAAGCGATCATACCCAGAG
	R:CTGCCTGAAGTCATCCACACTC
Claudin-1	F:TGGCTTCTCTGGGATGGATCGG
	R:CCTGAGCGGTCACGATGTTGTC
MUC-2	F:ATCCTCGACGCCTGTGACCTC
	R:GCTGCCCGCTGATGAAGTGAC
ZO-1	F:CCATTCAGGTCGCTCGCATGAC
	R:CATTGCTGGGCTGCTGTGGAG
IL-1β	F:GCAACTGTTCCTGAACTCAACT
	R:ATCTTTTGGGGTCCGTCAACT
IL-6	F:TAGTCCTTCCTACCCCAATTTCC
	R:TTGGTCCTTAGCCACTCCTTC
TNF-α	F:CCCTCACACTCAGATCATCTTCT
	R:GCTACGACGTGGGCTACAG
MyD88	F:ATCGGCTTAAGTTGTGTGTG
	R:AATCGTCAGAAACAACCACC
NF-κB	F:TAACAGCAGGACCCAAGGAC
	R:AGCCCCTAATACACGCCTCT
TLR-4	F:ACCTGGAATGGGAGGACAAT
	R:GTCCAAGTTGCCGTTTCTTG
GPR39	F:ATACCTGCGTAACCCTGATGG
	R:CATGTTCCTGAATTTGAGGTGGC
NHE3	F:TTGGCCGCCTTCTTATTCTGG
	R:TGAAAAGCAGGACAAGGAAATCT
CFTR	F:CTGGACCACACCAATTTTGAGG
	R:GCGTGGATAAGCTGGGGAT

Supplementary Table S3. Primer set for virulence factors analysis

Gene	5'–3' Primer sequence
gapA	F:CGTTAAAGGCGCTAACTTCG
	R:ACGGTGGTCATCAGACCTTC
eltA	F:TTGGTGATCCGGTGGGAAAC
	R:AGGAGGTTTCTGCGTTAGGTG
eltB	F:CACGGAGCTCCCCAGACTAT
	R:GCCTGCCATCGATTCCGTAT
estB	F:TGCCTATGCATCTACACAA
	R:CTCCAGCAGTACCATCTC
luxS	F:CAGTGCCAGTTCTTCGTTGC
	R:TGAACGTCTACCAGTGTGGC
bssS	F:TCCCTTCCTGCTCGGACTTA
	R:CAGACTCATCCGCTCGTAGG
tnaA	F:CGCCAAGAAAGATGCGATGG
	R:CGTCATACAGACCTACCGCC
motA	F:TGAACGACCCCCATTACAGC
	R:AGCGGTCACATGAACACCTT
faeG	F:ACTCAGAAAACCTGATGGTGAAACT
	R:CCCCACCTCTCCCTAACACA
